# Caveolin-1 Endows Order in Cholesterol-Rich Detergent Resistant Membranes

**DOI:** 10.3390/biom9070287

**Published:** 2019-07-17

**Authors:** Carla Raggi, Marco Diociaiuti, Giulio Caracciolo, Federica Fratini, Luca Fantozzi, Giovanni Piccaro, Katia Fecchi, Elisabetta Pizzi, Giuseppe Marano, Fiorella Ciaffoni, Elena Bravo, Maria L. Fiani, Massimo Sargiacomo

**Affiliations:** 1 National Center for Control and Evaluation of Medicines, Istituto Superiore di Sanità, 00161 Rome, Italy; 2 National Center for Rare Diseases, Istituto Superiore di Sanità, 00161 Rome, Italy; 3 Department of Molecular Medicine, “La Sapienza” University, 00161 Rome, Italy; 4 Scientific Service for Core Facilities, Istituto Superiore di Sanità, 00161 Rome, Italy; 5 ARPALAZIO, Via Salaria per L’Aquila 6/8, 02100 Rieti, Italy; 6 Notified Body, Istituto Superiore di Sanità, 00161 Rome, Italy; 7 Reference Centre for Gender Medicine, Istituto Superiore di Sanità, 00161 Rome, Italy; 8 Scientific Service for Research Coordination and Support, Istituto Superiore di Sanità, 00161 Rome, Italy; 9 National Center for Global Health, Istituto Superiore di Sanità, 00161 Rome, Italy

**Keywords:** caveolae, membranes/physical chemistry, lipid rafts, membranes/fluidity, membranes/model lipid-rafts, cholesterol-rich microdomains, liquid order, membrane heterogeneity, Langmuir films, X-ray diffraction

## Abstract

Cholesterol-enriched functional portions of plasma membranes, such as caveolae and rafts, were isolated from lungs of wild-type (WT) and caveolin-1 knockout (Cav-1 KO) mice within detergent resistant membranes (DRMs). To gain insight into their molecular composition we performed proteomic and lipid analysis on WT and Cav-1 KO-DRMs that showed predicted variations of proteomic profiles and negligible differences in lipid composition, while Langmuir monolayer technique and small and wide-angle X-ray scattering (SAXS-WAXS) were here originally introduced to study DRMs biophysical association state. Langmuir analysis of Cav-1 containing DRMs displayed an isotherm with a clear-cut feature, suggesting the coexistence of the liquid-ordered (*L_o_*) phase typical of the raft structure, namely “cholesterol-rich *L_o_* phase”, with a phase fully missing in Cav-1 KO that we named “caveolin-induced *L_o_* phase”. Furthermore, while the sole lipid component of both WT and KO-DRMs showed qualitatively similar isotherm configuration, the reinsertion of recombinant Cav-1 into WT-DRMs lipids restored the WT-DRM pattern. X-ray diffraction results confirmed that Cav-1 causes the formation of a “caveolin-induced *L_o_* phase”, as suggested by Langmuir experiments, allowing us to speculate about a possible structural model. These results show that the unique molecular link between Cav-1 and cholesterol can spur functional order in a lipid bilayer strictly derived from biological sources.

## 1. Introduction

The initial discovery that important functional plasma membrane proteins, such as GPI anchored protein, caveolin-1 (Cav-1), and signal transduction proteins, could be isolated within restricted microdomains resistant to Triton X-100 solubilization was, since the beginning, connected to the hypothesis of the existence of lipid rafts in biological membranes [1,2]. Detergent-resistant membranes (DRMs) enriched in Cav-1 were soon after characterized at the molecular level [1,3,4]. The original definition of lipid rafts as heterogeneous, highly dynamic, sterol, and sphingolipid-enriched domains that compartmentalize functional proteins [5], and possess a lipid structure that is equivalent to the liquid-ordered (*L_o_*) phase of model membranes [6], is nowadays insufficient to explain the many cellular roles attributed to lipid rafts [7,8]. In these last years there has been a revitalized interest in lipid rafts; however, despite recent advances in biochemical and microscopy approaches allowing light to be to shed on lipid organization and membrane heterogeneity in model membranes, the study of ordered membrane domains in vivo remains elusive. In cells at physiological temperatures, no visible membrane formation can be evidenced except for caveolae, although some indirect observation of small and dynamic nanostructures at 37 °C, presumably rafts, have been made [9]. Caveolae are structurally recognizable invaginations of the plasma membrane, whose formation is dependent on Cav-1 expression, a 22 kDa protein which is inserted into membranes in a cholesterol-dependent manner and is mostly localized on the cytoplasmic face of caveolae [10,11]. In addition to its structural role in promoting surface organization, Cav-1 serves as lipid binding adaptor protein, also exerting a pivotal role in membrane trafficking and signaling regulation, whose dysfunction is a cause of several disease conditions [12]. Absence of Cav-1 affects membrane organization and cell signaling, as this increases lipid components mobility and decreases plasma membrane ordered domains [13,14,15], supporting the hypothesis that caveolae represent a *L_o_* depository formed by specific lipids that stabilize and regulate plasma membrane fluidity [16]. 

Studies on models such as micelles, liposomes, and Langmuir film monolayers show that lipid mixtures containing cholesterol have the tendency be separated into coexisting *L_o_* and liquid-disordered (*L_d_*) phases, whose arrangements seem essential for plasma membrane functions, as in rafts [17], or in more organized structures, such as caveolae. *L_o_* phase, formed in model membranes enriched in cholesterol, as the result of tight acyl chain packing, is also resistant to detergent (TX 100) solubilization at 4 °C, whereas *L_d_* domains, poor in cholesterol and loose in fatty acids association, are susceptible to TX-100 solubilization [18]. As such, DRMs composed of coalescent lipids and proteins, thus consistently different from model membranes, may in fact generate thermodynamic unexplored lipid phases coexistence.

In the present study, we carried out a comparison between DRMs derived from lungs of wild-type (WT) and Cav-1 knockout (Cav-1 KO) mice to highlight the complex interactions underlying DRMs protein–lipid assembly. To this purpose we biochemically dissected DRMs into their protein and lipid building blocks.

We first analyzed proteomic and lipid profiles of WT and Cav-1 KO-DRMs that showed negligible differences for lipids and predicted differences in protein expression. To gain new insight into WT and KO-DRMs molecular associations we set off biophysical studies using the Langmuir monolayer technique coupled with the small and wide-angle X-ray scattering (SAXS-WAXS) technique. Langmuir film monolayers have been widely used to study the thermodynamics of the aggregation of lipids simulating a cellular membrane and its interaction with several proteins [19,20], and more recently applied to lipid rafts investigations [21,22]. Furthermore, we have previously shown, by X-ray diffraction at low hydration, the presence of two distinct phases in Cav-1 DRMs that coexist with segregated cholesterol crystallites [23].

In the present paper, we report Langmuir compression results, which displayed in WT-DRMs an isotherm with a clear-cut feature representing a *L_o_* phase fully missing in Cav-1 KO. Notably, this feature was located in the pressure range of the isotherm where the equivalence in physical behavior between mono- and bilayers is known [24], suggesting that this *L_o_* phase that we named “caveolin-induced *L_o_* phase” is characteristic of the actual cellular membranes. Moreover, while the sole lipid component of both WT and KO DRMs showed an identical isotherm configuration, the reinsertion of recombinant Cav-1 into DRMs lipids completely restored the WT-DRMs pattern, suggesting that the unique molecular link between Cav-1 and cholesterol can spur functional order in a lipid bilayer strictly derived from biological sources.

Looking for this ordered phase, we expanded our previous X-ray scattering observations on lipid bilayers by comparing WT and KO-DRMs. Notably, X-ray results clearly showed that WT solid-supported DRMs contained exclusive ordered structures that were completely absent in Cav-1 KO-DRMs.

## 2. Materials and Methods

### 2.1. Animals

Procedures involving animals and their care were conducted in conformity with the institutional guidelines that are in compliance with national and international laws and policies (Legislative Decree no. 116/92 and Directive 86/609/EEC), and were approved by the Italian Ministry of Health (code no. 219/2010-B released on 1/12/2010). Animal welfare was routinely checked by veterinarians from the Service for Biotechnology and Animal Welfare. Male Cav-1^−/−^ mice (JacK Laboratories, Bar Harbor, Maine, USA) and male C57BL/6 mice (Harlan, San Pietro al Nanisano, Italy) 12 weeks old were used for experiments. Since Cav-1^−/−^ mice have a mixed 129/Sv*C57BL/6J*SJL genetic background, we mated cav1^−/−^ mice with C57BL/6 mice to produce F1 mice heterozygous for both knock out genes. Thereafter, F1 heterozygous mice were mated to produce F2 wild-type (WT) and Cav-1^−/−^ (KO) mice. Genotyping was performed on tail DNA using standard protocols [25].

### 2.2. Antibodies

Anti-Cav-1 and anti-cavin-1 polyclonal antibodies were purchased from Santa Cruz Biotechnology (Santa Cruz, CA, USA) and Abcam (Cambridge, UK), respectively. Anti-Cav-2 and anti-flotillin-1 (Flot-1) monoclonal antibodies were obtained from BD Transduction Laboratories (Lexington, UK).

### 2.3. DRM Extraction

Isolation of caveolae and DRM domains from lung and visceral adipose tissue was performed as previously described [3] with minor modifications. Approximately 200 mg of lung or visceral adipose tissue, from WT and KO mice, were removed and washed in phosphate buffer saline (PBS) solution, lysed with a Polytron homogenizer for 30 s, and homogenized on ice in 0.75 mL MES buffer (25 mM 2-[N-morpholino] ethanesulfonic acid (MES) pH 6.5, 150 mM NaCl) containing 1% TX-100 and a protease inhibitor cocktail (Roche). The extract was adjusted to 40% (*w*/*v*) sucrose, transferred to an ultracentrifuge tube, overlaid with 1.5 mL of 30% (*w*/*v*) sucrose and 1.5 mL 5% (*w*/*v*) sucrose in MES buffer, and centrifuged at 100.000× *g* for 16 h at 4 °C in a Beckman SW60 rotor. Twelve 0.375 mL fractions were collected and fractions 3–6 from low-buoyant density were pooled. Five volumes of MES buffer were added to pooled fractions and centrifuged for 30 min at 4 °C at 30,000× *g*. Typically 0.2–0.3 mg of DRMs were recovered.

### 2.4. Protein Determination

Protein content was quantitated using the BCA Protein Assay Kit (Pierce Biotechnology, Rockford, IL, USA).

### 2.5. Western Blot Analysis

Equal volumes of each sucrose gradient fraction were run on 10% SDS-PAGE gels and transferred to nitrocellulose membranes. Membranes were blocked for 1 h in 10 mM Tris-HCl (pH 8.0), 150 mM NaCl, 0.1% Tween 20 (TBST) containing 5% powdered skim milk. Membranes were incubated for 1 h with the primary antibody in TBST followed by secondary antibodies horseradish peroxidase conjugated and visualized using an enhanced chemiluminescence kit (Pierce, Rockford, IL, USA).

### 2.6. Proteomic Analysis

DRM proteins (20 μg) were solubilized in NuPage lithium dodecyl sulfate (LDS) sample buffer (Invitrogen) at 70 °C for 1 h, separated on Invitrogen precast 4%–20% Bis-Tris gels and stained with Comassie Colloidal Blue. Gel lanes were cut into 18–20 sequential slices about 4 mm wide.

In-gel tryptic digestion was performed as already described [26] with slight modifications. Briefly, gel slices were destained in a mixture 1:1 of acetonitrile (ACN)/50 mM ammonium hydrogen carbonate (AHC); protein cysteines were reduced with 10 mM DTT/25 mM ACH (30 min at 56 °C) and alkylated by 55 mM Iodoacetamide/25 mM ACH (30 min in the dark at RT). Gel slices were then shrunken in ACN and rehydrated for 40 min on ice with a solution of 12.5 ng/μL of sequence grade bovine trypsin/50 mM ACH and protein digestion was carried out overnight at 37 °C.

Reverse- phase liquid-chromatography tandem mass spectrometry (RP-LC-MS/MS) was performed on an LTQ XL linear ion trap coupled on line to a Dionex Ultimate 3000. Trypsin digests were desalted in a trap column and then separated in a reverse phase column, a 10 cm long fused silica capillary (Silica Tips FS 360 × 75 × 8 μm, New Objective, Woburn, MA, USA), slurry-packed in-house with 5 μm, 200 Å pore size C18 resin (Michrom BioResources, CA, USA). Peptides were eluted using a linear gradient from 96% A (5% ACN, 0.1% FA) to 60% B (95% ACN, 0.1% FA) in 60 min, at 300 nL/min flow rate.

The LTQ operated in a data-dependent mode in which each full MS scan event was followed by five MS/MS scans, where the five most abundant parent ions were dynamically selected and fragmented with collision induction dissociation (CID) using a collision energy of 35%. Tandem mass spectra files were analyzed by Sequest HT search engine with Proteome Discoverer 1.4 (ThermoFisher) as already described [26]. Mouse Uniprot reviewed database (released 2014) was used. The carbamidomethylation of cysteines was specified as fixed modification, while the oxidation of methionine and phosphorylation of serine, threonine, and tyrosine were set as variable modification. The percolator tool was used for peptide validation based on the q-value and high confidence was chosen, corresponding to a false discovery rate ≤ 0.01 on peptide-level. Proteins were identified with a minimum of 2 peptides rank = 1, and peptides must achieve a minimum cross correlation score of 1.8 for [M + H]^1+^, 2.5 for [M + 2H]^2+^, 3 for [M + 3H]^3+^. Protein and peptide identification data for WT and Cav-1 KO are reported, respectively, in Supplementary Tables S1 and S2. The identified proteins were grouped into classes based on Panther Protein Class annotation (version 12).

### 2.7. Lipid Analysis

0.1 mg of DRMs (calculated for protein) and equal volumes of each sucrose gradient fraction were used for lipid extraction by chloroform:methanol:water (2:1:0.15 *v*/*v*) solution according to Folch [27]. Lipid extracts were dried under nitrogen gas, reconstituted in chloroform:methanol (2:1, *v*/*v*), and analyzed by high performance thin layer chromatography (HPTLC) on HPTLC plates (Silica Gel 60, 0.20-mm layer thickness) from Merck (Darmstadt, Germany). Neutral lipids were separated in hexane:diethyl ether:acetic acid (70:30:1, *v*/*v*), and phospholipids in chloroform:methanol:acetic acid:formic acid:water (35:15:6:2:1,*v*:*v*). Lipid spots were visualized by copper acid (3% *w*/*v*) staining. The plate was heated for 5 min at 180 °C and the amount of each lipid was determined by densitometry relative to its standard using the imaging densitometer GS-700 (Bio-Rad). Standards of phospholipids (phosphatidylcholine (PC); phosphatidylethanolamine (PE)), sphingomyelin (SM), triglyceride (TG), and cholesterol (Chol) were purchased from Sigma Aldrich Co. (St. Louis, Missouri, USA). Analysis of the fatty acid methyl esters of the single phospholipid classes was performed as already described with minor modifications [28]. Briefly, the fatty acid methyl esters were prepared by acid catalyzed trans-esterification [29] using heptadecanoic acid as internal standard, extracted in hexane and evaporated under nitrogen gas. Then samples were analyzed by gas liquid chromatography in a Hewlett-Packard 5890 Series II gas chromatograph equipped with a flame ionization response detector, a capillary column Omega wax M 320 (30 mU, 0.32 mm), and a 0.25 mm film (Supelco, Inc.). A temperature gradient program was used and calibration was performed with the reference mixture ME-64 (Larodan Fine Chemicals, Malmo, Sweden) and a polyunsaturated fatty acids (PUFA-2) standard mixture (Supelco, Inc.).

### 2.8. Cav-1 Insertion in Liposomes Derived from DRMs

Lipids from WT and Cav-1 KO-DRMs (0.1 mg of protein) were extracted according to Folch [27] by mixing with chloroform, methanol, and water to reach final ratios of chloroform:methanol:water (2:1:0.15 *v*:*v*). Lipid extracts were dried under nitrogen, solubilized in sample buffer, loaded on SDS-PAGE gels, and stained with Silver solution to verify the absence of proteins. Recombinant Histidine-Tagged Cav-1 previously used for proteoliposomes formation [11] was obtained from Primm Pharma S.r.l. (San Raffaele Biomedical Science-Milan, Italy). Purified Escherichia coli (*E. coli*) Cav-1 was dialyzed against MES buffer for 1 h at 4 °C. Cav-1 and WT DRM lipid extract, in the ratio 1:2 *w*/*w* (same ratio as in WT DRM), were vigorously mixed at 25 °C in MES buffer and sonicated in an ultrasonic bath for 30 min at 37 °C. Newly formed proteoliposomes were recovered by 30.000× *g* centrifugation for 30 min at 4 °C in microcentrifuge and analyzed by Western blot for Cav-1 (Supplementary Figure S1).

### 2.9. Langmuir Measurements

To prepare DRM-derived monolayers aliquots of WT-DRMs, Cav-1 KO and Cav-1 containing liposomes were lyophilized and then dissolved in chloroform:methanol:water (2:1:0.15 *v*:*v*). To prepare monolayers mimicking lipid rafts, dipalmitoylphosphatidylcholine (DPPC) dissolved in pure chloroform (1 mg/mL) was mixed with monosialotetrahexosylganglioside (GM1) and cholesterol solutions (1 mg/mL) at a molar fraction of 49/4/47 mol.% and dissolved in chloroform:methanol:water (2:1:0.15 *v*/*v*). All mixtures were finally deposited on Langmuir trough and analyzed. Surface pressure-area (π-A) isotherm studies were carried out using KSV Minitrough Langmuir apparatus (KSV, Helsinki, Finland), placed on an anti-vibration table and enclosed in a box to avoid contamination and dust deposition. The surface tension (π) of the lipid monolayer was measured using the Wilhelmy method, using a roughened platinum plate, with an accuracy of 1 mN/m [24]. All the experiments were performed with thermostated trough by a water-bath circulator (C25, Haake, Kerlsruhe, Germany) at a temperature of 25 ± 0.1 °C with Langmuir monolayers spread on a PBS (0.15 M) subphase at pH 7.4. Such a subphase was chosen after a large number of experiments aimed at achieving a good film stability and optimal reproducibility. Compression was achieved with the symmetric movement of the two opposing barriers. Trough and barriers were thoroughly cleaned before each measurement with appropriate solvents, and rinsed with ultrapure water. Prior to film deposition, the surface was cleaned repeatedly by sweeping the barriers and aspirating the surface in between, until no change in surface pressure was detectable when comparing the closed and open positions. Monitoring changes in the surface pressure and the mean molecular area during film compression was done using the Wilhelmy method by a roughened platinum plate. Appropriate amounts of the solutions (30–100 μL) were spread in a drop-wise manner with a 25 μL microsyringe. Monolayers were compressed at a rate of 30 cm^2^/min. Three successive compression–expansion cycles in the *L_d_* phase of the monolayer were performed to check monolayers stability and to verify the absence of hysteresis, before starting the regular compression run. All isotherms (at least before the collapsed phase region) presented were ensured to be reproducible and the measurements for each isotherm were carried out more than three times for each sample. Several samples were studied and reported isotherms were obtained as the average of at least five independent preparations.

### 2.10. X-Ray Scattering Experiments

DMR extract was not solubilized in organic solvent, but fresh DMRs were used. DMRs consist of independent scattering domains that are isotropically distributed in space. Such powder-like samples have a low scattering power and the scattered intensity rapidly falls off with increasing transfer momentum q. Conversely, solid-supported highly-aligned multilayers preserve structural information and give more intensity for higher orders diffraction peaks, thus allowing for more accurate diffraction analyses. To take advantage of sample orientation, 100 μL of the solution containing fresh DMRs were deposited onto the freshly-cleaved surface of oriented <100> silicon wafers and dehydrated under a gentle nitrogen flux. This procedure results in the formation of a dehydrated multilayer structure made of aligned DMRs with preferential orientation along the normal to the solid support [23]. To extend all the retrieved structural information to the biologically-relevant excess water condition, solid-supported dehydrated samples were transferred in a humidity chamber that allows aligned multi-bilayer systems to be fully hydrated [30]. X-ray diffraction experiments were carried out using an X-ray diffractometer elsewhere described [23,31]. Both SAXS and WAXS scans were collected at room temperature for t = 100 s and no evidence of radiation damage was detected.

### 2.11. Statistical Analysis

Student’s *t*-test was used for the analysis of differences between samples (GraphPad Prism version 7.00 software).

## 3. Results

### 3.1. Characterization of DRMs from Lung Tissue of WT and Cav-1 KO Mice

**Figure 1:**
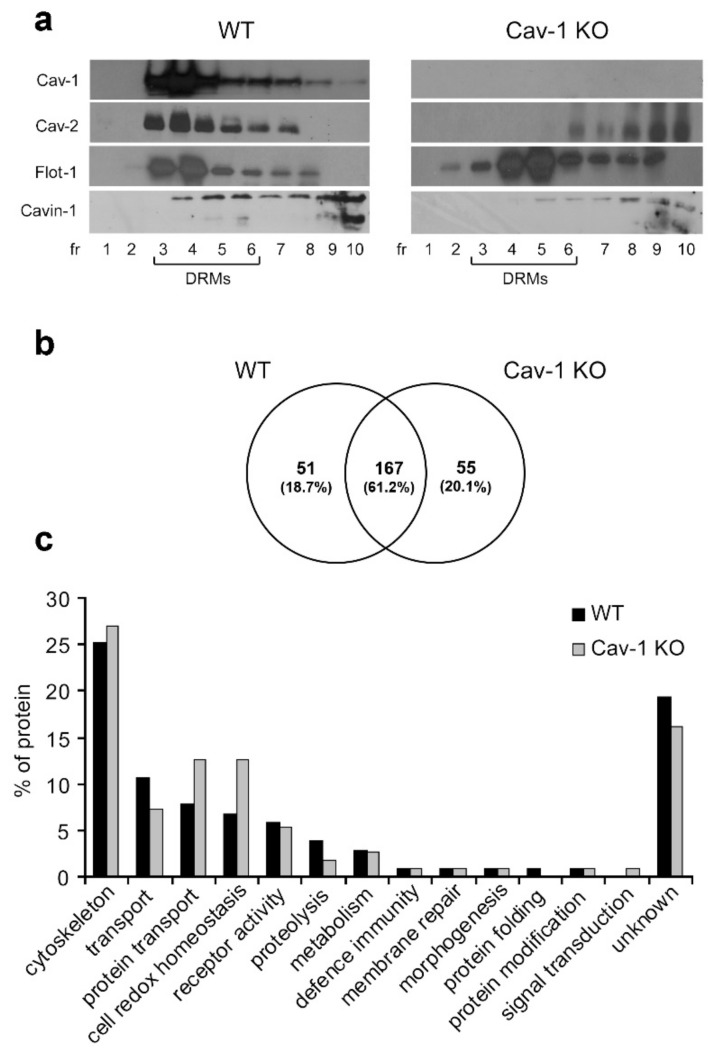
Characterization of detergent-resistant membranes (DRMs)-associated proteins. (**a**) Equal volumes of sucrose gradient fractions from lungs of wild-type (WT) and caveolin-1 knockout (Cav-1 KO) mice were subjected to western blot analyses for Cav-1, Cav-2, Flot-1, and Cavin-1. (**b**) Comparative analysis by reverse phase liquid chromatography-tandem mass spectrometry (RP-LC-MS/MS) of DRMs (pooled fractions 3–6). The Venn diagram shows the number of specific and common WT/Cav-1 KO proteins identified in at least two of the three independent biological replicates. (**c**) Classification of WT and KO DRM proteins into functional categories based on PANTHER v.12.

Lung tissue, composed mostly of Type I pneumocytes and endothelial cells, is a known rich source of caveolae and thus was used for DRMs extraction. Sucrose gradient fractions obtained from lung tissue treated with 1% TX 100 from WT and Cav-1 KO mice were analyzed by Western blot to determine the distribution of the caveolae marker Cav-1 and the lipid raft marker Flot-1 here used as KO DRM marker. As shown in Figure 1a, Cav 1 colocalizes with Cav-2 [32], and with cavin-1 [33] in TX 100 insoluble fractions 3–6 (corresponding to DRMs) of WT lungs whereas in KO samples Cav-2 and cavin-1 redistribute in the TX 100 soluble fractions 7–12.

To characterize protein profiles, WT and Cav-1 KO-DRMs were subjected to SDS/PAGE separation. Protein bands were excided from the gel, digested with Trypsin, and analyzed by RP LC MS/MS. We considered proteins identified with at least two peptides in at least two of the three biological replicates analyzed. According to these criteria, we detected a total of 219 proteins in WT and 223 in KO-DRMs, of which 167 proteins were common to both samples and 51 and 55 proteins (about 20%) were specific for WT and KO, respectively (Figure 1b). The complete list of proteins is provided in Supplementary Table S1.

We performed a functional analysis of the two proteomes according to Gene Ontology Panther v.12 annotation. Proteins were grouped in 22 biological classes. Results in Figure 1c show that the differences between the two proteomes mainly concern proteins involved in the oxidoreductase process that are more abundant in KO-DRMs, and proteins involved in actin binding, cell junction, and G-protein modulator that are more represented in WT-DRMs (Supplementary Table S1).

### 3.2. Lipid Analysis

**Figure 2:**
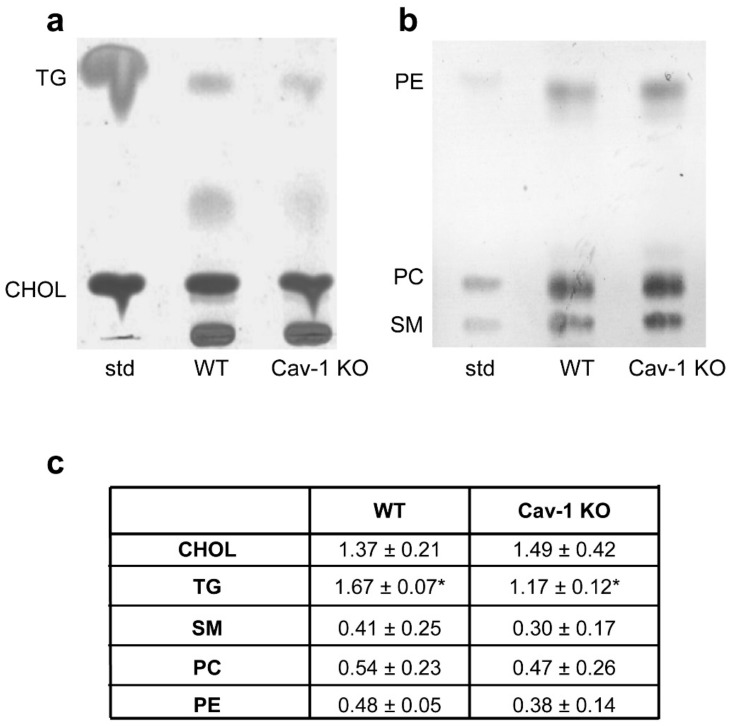
Characterization of DRM lipids. Post-nuclear homogenates from WT (**a**) and Cav-1 KO (**b**) lung tissue were extracted using 1% TX-100 and fractionated in 5%–30% sucrose gradient. Equal volumes of sucrose gradient fractions were subjected to high performance thin layer chromatography (HPTLC) analysis. Fractions 3–6 corresponding to DRMs showed enrichment of sphingomyelin (SM) and cholesterol (CHOL). (**c**) HPTLC chromatograms of lipids extracted from DRMs. 10 µg of each lipid standard were spotted on the plate. Cholesterol (CHOL) and triacylglycerols (TG) analysis. Phospholipids (PE; PC) and sphingomyelin (SM) analysis. Lipid concentrations were normalized to DRM protein and calculated against the reference standard. Data are presented as mean ± s.e. (*n* = 3) (* *p* < 0.05).

To define the lipid profile distribution of lung TX 100 extracts, sucrose gradient fractions were subjected to TLC analysis. An enrichment in phospholipids, sphingomyelin and cholesterol was clearly visible in both WT and KO fractions 3–6 that correspond to DRMs (Supplementary Figure S2). Lipid content of DRMs (Figure 2a,b) was then analyzed by densitometry following TLC chromatography (Figure 2c,d).

No statistically significant variation was observed in cholesterol, phospholipids (PC, PE) and sphingomyelin (SM) concentration in WT and KO-DRMs (Figure 2C). These data match previous studies on WT and KO mouse embryonic fibroblasts (MEF), in which absence of Cav-1 did not alter membrane cholesterol yet differ in glycerophospholipids profiles that in our case were unchanged [15,34]. Such discrepancy may be due to the fact that our lipid analysis was performed on whole lung tissue as opposed to MEF primary cell cultures. Notably, we found a TG increase in WT with respect to KO-DRMs explained by the fact that caveolae is a major plasma membrane site of triglycerides synthesis [35]. We then evaluated acyl chain composition and unsaturation index of phospholipid classes by gas chromatography. As can be seen in Supplementary Table S2, there was no significant difference in the percentages of saturated, monounsaturated, and polyunsaturated fatty acids of PC, PE, and sphingomyelin species.

### 3.3. Langmuir Films

To investigate the differences induced in the monolayer by the presence of Cav-1, we performed a thermodynamic study using the Langmuir technique. DRMs from WT and Cav-1 KO mouse were used to prepare Langmuir monolayers.

**Figure 3:**
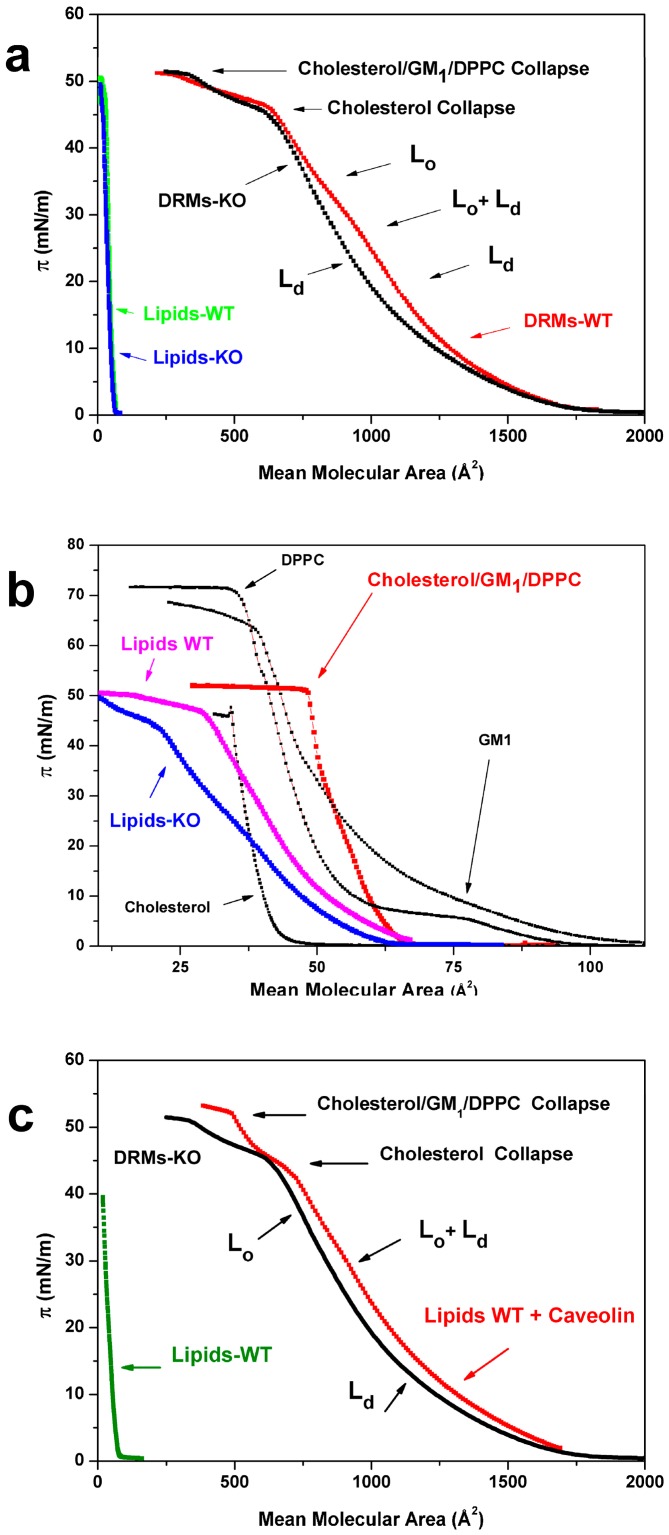
DRMs compression isotherms. (**a**) Compression isotherms of Langmuir films prepared with DRMs of WT (red) and Cav-1 KO (black) mice and isotherms relative to the same samples after protein removal (green and blue). (**b**) Protein-free DRMs isotherms (WT pink and KO blue) in comparison to the isotherm of a simplified “raft” model made of cholesterol, monosialotetrahexosylganglioside (GM1), and dipalmitoylphosphatidylcholine (DPPC) (red) and to the single lipid components (black). (**c**) The reconstitution of Cav-1 in proteoliposomes: Isotherm obtained after reconstitution (red) in comparison with the KO-DRMs isotherm (black) and the protein-free sample obtained from WT-DRMs, used to prepare proteoliposomes (green). *L_o_*, liquid-ordered phase; *L_d_*, liquid-disordered phase; GM1, monosialotetrahexosylganglioside; DPPC, dipalmitoylphosphatidylcholine.

Figure 3a shows the π-mean molecular area (MMA) compression isotherms of WT and Cav-1 KO-DRMs (DMRs-WT and DRMs-KO), together with isotherms of the same samples deprived of the protein component (Lipids-WT and Lipids-KO). For what concerns the WT and Cav-1 KO isotherms, some general considerations follow: Both isotherms were characterized by the same collapse pressure of about 52 mN/m, reached at about MMA = 260 Å^2^ and a common and strong inflection at π = 45 mN/m reached at about MMA = 650 Å^2^. Lastly, curves overlapped at MMA values higher than 1500 Å^2^. As it can be observed, the curves of the protein-free samples, made of the plain lipid components, are practically superimposed and located at very low MMA values due to the low mean molecular weight assigned to lipids when compared to the DRM one (1000 vs. 10,000 for the whole DRMs). When depicted with an expanded x-axis (Figure 3b Lipids-WT pink and Lipids-KO blue), the compression isotherms relative to the two lipid-free samples appeared qualitatively similar to each other. The small differences could be attributed to the actual lipid concentrations obtained after the protein extraction and to the higher TG content in the WT-DRMs (Figure 2c). However, from a qualitative point of view, the two isotherms were both characterized by a collapse pressure of about 50 mN/m, an evident shoulder around 45 mN/m, and to reach zero around 60 Å.

With the aim to identify the role played by the main components constituting the lipid fraction of DRMs, we report in the same figure isotherms relative to a mixture made of cholesterol, DPPC, and GM1 (0.47; 0.49; 0.04), simulating lipid-raft, together with the isotherms of the plain components. The curves of the protein-free and the model sample are obviously different from each other, due to the oversimplification of the mixture, where only one lipid (DPPC) and ganglioside (GM1) are present. However, we noted that the collapse pressure of the mixture was about π = 52 mN/m, which is very similar to the collapse pressure of our protein-free samples and different from the collapse pressures of the plain components. We also noted that both DRM isotherms (Figure 3a) showed a collapse pressure value (52 mN/m) of the lipid-free samples and of the lipid-raft simplified model. This observation strongly suggests that the DRM film matrix was composed by the same lipid components. Moreover, we also noted that both protein-free isotherms (Figure 3b) were characterized by a marked shoulder, located at the cholesterol collapse pressure value (π = 45 mN/m). This suggests that the high levels of cholesterol, typical of the DRMs, induced a plateau in the curve that can be interpreted as due to the coexistence of a “cholesterol-rich *L_o_* phase” with the *L_d_* phase typical of unsaturated lipids. Notably, this feature was also evident in the isotherms relative to DRM of both WT and Cav-1 KO, suggesting in these samples the presence of a “cholesterol-rich *L_o_* phase”, due to the high level of cholesterol. As far as the collapse zone is concerned, the isotherms relative to DRM of WT and Cav-1 KO samples did not show relevant differences. Conversely, a big difference in the curves of Figure 3a occurred around MMA = 900 Å^2^, where the WT isotherm showed a feature completely absent in the KO curve. It is well known that the occurrence of a plateau or a shoulder in the isotherm represents the coexistence at that pressure of the two phases [24] and that exponential decreasing curves can be associated with *L_d_* phases (sometimes called liquid-expanded) whereas linear behaviors associated to *L_o_* phases (more in general liquid-condensed) [24,36,37]. Based on this consideration, we speculate that the characteristic feature, occurring only in the WT-DRMs isotherm (red curve in Figure 3a), can be ascribed to a *L_o_* phase. The complete lack of this feature in the Cav-1 KO-DRMs isotherm suggest that this phase was induced, directly or indirectly, by the presence of Cav-1 in the DRMs film, named “caveolin-induced *L_o_* phase”. In order to verify this hypothesis, we reconstituted purified Cav-1 with lipids extracted from WT-DRMs (proteoliposomes) (Supplementary Figure S1) and compared them with the KO-DRMs sample. The resulting compression isotherms are reported in Figure 3c. It is interesting to note that the curve relative to the reconstituted sample looks like the one relative to the WT sample of Figure 3a. Note that the reconstituted curve is very different from the isotherm relative to the KO-DRMs, with particular attention to the typical feature of the WT located around 900 Å^2^. For comparison, we also report the isotherm of the protein-free sample obtained from WT-DRMs, used to prepare the reconstituted sample. On the base of available knowledge, our results strongly suggest that the characteristic feature relative to the *L_o_* phase in the WT sample, named “caveolin-induced *L_o_* phase”, occurring at a compression pressure of about 30 mN/m, can be attributed to the effects induced by Cav-1 in the Langmuir monolayer structural organization. We note that this feature was obtained where the equivalence in the physical behavior between mono- and bilayers is known [24], suggesting that the responsible molecular arrangement can be formed in the both cases.

### 3.4. Small and Wide-Angle X-Ray Scattering

**Figure 4:**
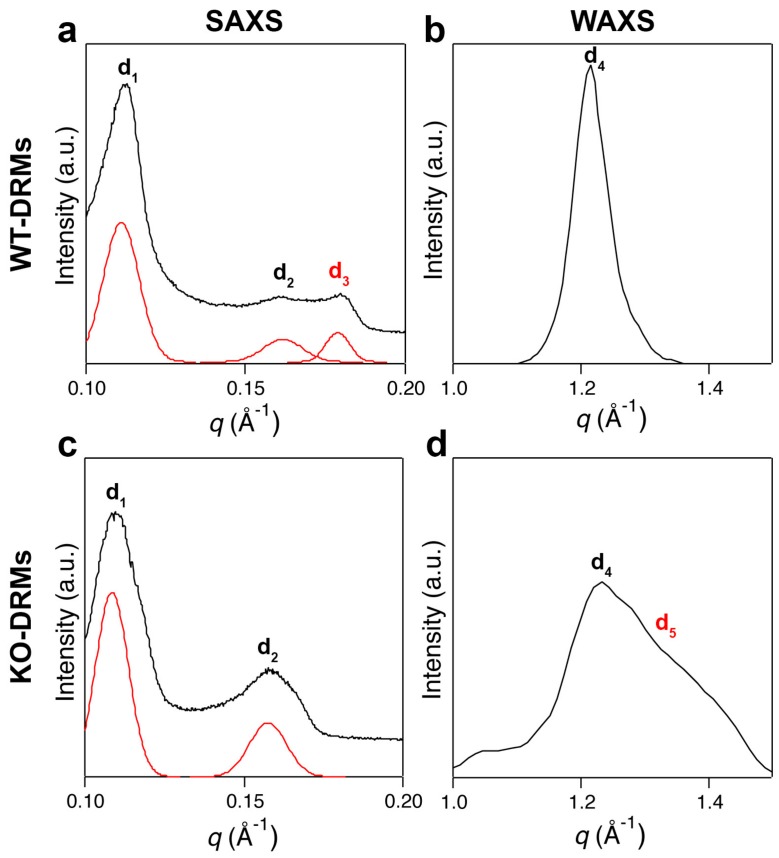
X-ray scattering experiments. (**a**) Small angle X-ray scattering (SAXS) pattern of lung Cav-1 WT-DRMs. The first two Bragg peaks are the first-order reflections of two lamellar phases with d-spacings of d_1_ = 57.2 and d_2_ = 39.9 Å. A third diffraction peak, with lamellar periodicity of d_3_ = 34.0 Å (symbol in read), is also present. (**b**) The wide-angle X-ray scattering (WAXS) pattern of lung WT-DRMs shows a sharp diffraction peak, characteristic of an ordered lipid phase with a packing spacing of d_4_ = 4.2 Å. (**c**) The SAXS pattern of lung KO-DRMs resembled that of Cav-1 WT-DRMs. However, the main difference is the total absence of the diffraction peak d_3_. (**d**) Conversely, in the WAXS scan of lung KO-DRMs another contribution appears at d_5_ = 3 Å, together with the less intense peak relative to d_4_ = 4.2 Å. Double peak is characteristic of disordered lipid phases.

We compared our previous findings on WT-DRMs [23] with the KO-DRMs counterparts to confirm the role of Cav-1 in promoting lipid order, as suggested from Langmuir studies in the pressure range of mono- and bilayer equivalence. Figure 4a shows the SAXS pattern of WT-DRMs Cav-1. Three Bragg peaks were detected that could not be indexed on the basis of any known lipid phase [38,39].

The first two diffraction peaks of Figure 4a were then interpreted as the first-order reflections of two lamellar phases with distinct bilayer thickness (d_1_ = 57.2 and d_2_ = 39.9 Å, respectively). The pattern shows a third peak, corresponding to a periodicity of d_3_ = 34 Å. Several reports on phospholipids–cholesterol mixtures have shown that, depending on the nature of the phospholipid and the history of the sample (temperature, hydration etc.), cholesterol crystallites can be in the form of cholesterol monohydrate, anhydrous cholesterol, or a mixture of both forms. Both the anhydrous and monohydrate forms of crystalline cholesterol contain the same pseudo bilayer structure with repeating distance of approximately 34 Å [40,41]. When the SAXS experiment was replicated with KO-DRMs, a remarkable differences was observed: The diffraction peak at d_3_ = 34 Å was totally lost (Figure 4c) and only two peaks were observed, relative to d_1_ = 58.1 and d_2_ = 40.4 Å. The small shift in the q-position of the two peaks, with respect to their counterpart in WT-DRMs, was most likely due to a little change in sample humidity. The presence of the third peak strongly suggests that Cav-1 induces the formation of a “caveolin-induced *L_o_* phase”, rich in cholesterol. For what concerns WAXS pattern relative to WT-DRMs (Figure 4b), a sharp and intense diffraction peak corresponding to a periodicity of d_4_ = 4.2 Å was observed. The same experiment performed on KO-DRMs gave a pattern (Figure 4b) where this sharp and symmetric peak was much less intense and a second contribution appeared on the right (d_5_ = 3 Å). This indicates that a more disordered arrangement occurred in the absence of Cav-1. The very similar phenomenology seemed to be rather general, since very similar SAXS and WAXS patterns were found in WT and KO-DRMs collected from the adipose tissue (Supplementary Figure S3).

## 4. Discussion

Caveolae morphology depends on Cav-1 close association with cholesterol, and it is corroborated by Cav-1 oligomerization that contributes to microdomains consistency and functionality [42]. Cav-1 preference for cholesterol-rich bilayers remains mostly unknown although an interesting hypothesis proposes that, rather than direct Cav-1-cholesterol binding, changes in membrane physical properties derived from the membrane ordered phases may occur [43,44].

We reasoned that as Cav-1, the major structural component for caveolae formation, is extremely concentrated in DRMs because of its strict association with cholesterol, it could constitute a native probe for the study of *L_o_* and *L_d_* phase coexistence within membranes. Due to the high complexity of the studied systems, we tried to focus on the role played by Cav-1 by comparing the physical properties of WT and Cav-1 KO-DRMs. The results presented showed proteomic profiles that differ by 20% in their components’ proteins, mainly concerning proteins involved in oxidoreductase process and proteins involved in actin binding, cell junction, and G-protein modulator. Previous studies obtained by down-regulating Cav-1 in endothelial cells with small interfering RNA, already indicated that the distribution of proteins normally associated with lipid rafts is unaffected by loss of Cav-1 expression [45].

We addressed a comparative study on lipid phase behavior of WT and Cav-1 KO-DRMs by Langmuir compression isotherms and X-ray scattering profile combined analysis. We would like to underline that Langmuir and X-ray scattering experiments were performed on different molecular systems: In the first case, a monolayer obtained at the water–air interface after the complete solubilization of the DRMs components; in the second, a native bilayer deposited directly onto a substrate and dehydrated. A wide range of film pressure was explored in the Langmuir film compression, ranging from gaseous to solid state, and not necessarily representative of a native biological system. However, it is well known that cellular bilayers are characterized by a tension of about 30 mN/m and that a comparison between bi- and monolayers can be performed around this value [24].

As far as the isotherms study is concerned, all analyzed samples were characterized by a collapse pressure typical of the mixture of DPPC, cholesterol, and GM1 used as a simplified model of the lipid rafts [46]. Moreover, in both WT and KO samples, compression isotherms shared a common feature located close to the collapse region. This feature can be interpreted, observing the isotherm of the plain cholesterol (Figure 3b), as a fingerprint of cholesterol and indicates the occurrence, in both samples, of a “cholesterol-rich *L_o_* phase”. This is in good agreement with literature data concerning the composition of lipid-rafts [45,46]. For what concerns the differences induced by Cav1, it is worth noting that only DRMs containing Cav-1 produced isotherms with the occurrence of second “caveolin-induced *L_o_* phases” (red curve of Figure 3a) while Cav-1 KO-DRMs isotherms expressed only a “cholesterol-rich *L_o_* phase”. This difference strongly suggests a pivotal role played by Cav-1 in endowing order in DRMs independently of the other DRMs proteins that were unchanged, as indicated by our proteomic results. Notably, the “caveolin-induced *L_o_* phase” occurring in the WT-DRMs is observed in our experiments at a compression value of about 30 mN/m (red curve of Figure 3a), supporting the idea that this protein exerts its action in the actual cellular bilayer. We note that when WT and Cav-1 KO-DRMs were deprived of proteins, from a qualitative point of view the two isotherms were very similar each other. Notably, both isotherms maintained the feature ascribed to a “cholesterol-rich *L_o_* phase”, which is typical of the lipid-rafts, even though small differences due to experimental factor and TG content can be observed. Our observations are in good agreement with compression isotherms obtained with lipid mixtures containing DPPC/cholesterol/GM1, used as a lipid-rafts model [47], showing the same collapse pressure (Figure 3b).

To further assess the effect induced on isotherms by the occurrence of Cav-1 in the rafts model, we reconstituted the purified protein Cav-1 in the lipid components of the WT sample. Notably, this reintroduction restored the formation of an isotherm similar to that relative to WT-DRMs, very different from the KO-DRMs one, characterized by the feature seen only in WT-DRMs at about 30 mN/m (Figure 3c) and attributed to a “caveolin-induced *L_o_* phase”. This strongly suggests that Cav-1 plays a pivotal role in endowing order in DRMs.

To structurally investigate DRM membrane structure and acyl chain order, at short and long range in an actual bilayer and thus at a tension of about 30 mN/m, X-ray scattering experiments were performed on deposited WT and Cav-1 KO-DRMs.

For what concerns SAXS, it allows for the detection of lamellar repeat spacings that are directly related to the thickness of lipid membranes. Since *L_o_* membranes are generally thicker than *L_d_* ones, detection of distinct repeat spacings in the SAXS scan is an effective tool to identify phase coexistence. The existence of two repeat spacings (d_1_ = 57.2 and d_2_ = 39.9 Å), as that observed in the SAXS patterns of both WT and KO scans reported in Figure 4, is a validated criterion for coexistence of *L_o_* and *L_d_* domains [48,49]. Interestingly, SAXS experiments performed on either lung (Figure 4) or adipose tissue (Supplementary Figure S3) DRMs domains showed evidence of a diffraction peak (d_3_ = 34 Å) only in the presence of Cav-1 that is WT-DRMs, lacking in the Cav-1 KO ones. This suggests that Cav-1 induces the constitution of a “caveolin-induced *L_o_* phase” in good agreement with our Langmuir results.

Chain ordering is one of the fundamental features that discriminate the different lamellar phases and WAXS is very useful because it directly probes chain–chain correlations [50]. In the WT WAXS spectrum (Figure 4b) an intense and narrow diffraction peak, unambiguously indicative of *L_o_* domains, was found (d_4_ = 4.2 Å). In contrast, this peak was much less intense in the KO spectrum (Figure 4d) and a second peak appeared on the left (d_5_ = 3 Å), merged with the first and forming a broad and asymmetric band, indicating the occurrence of a less ordered phase. This outcome further supports the hypothesis that Cav-1 endows order in the molecular arrangement.

**Figure 5:**
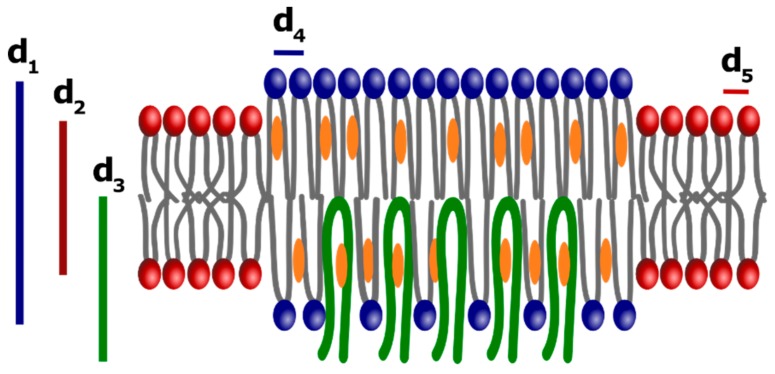
Schematic representation of the DRMs bilayer molecular organization. The body of our results, Langmuir isotherm and X-ray scattering experiments, suggest that an ordered phase made of saturated lipids (sphingolipids and gangliosides) and cholesterol is formed (blu), named “cholesterol-rich *L_o_* phase”, together with a more fluid *L_d_* phase made of unsaturated lipids (red). From X-ray scattering results, d_1_ = 57.2 and d_4_ = 4.2 Å could be associated with the “cholesterol-rich *L_o_* phase”. A less ordered *L_d_* phase, characterized by d_2_ = 39.9 and d_5_ = 3 Å, is also present. Both phases are typical of the lipid-rafts and are present even in the absence of Cav-1 (KO-DRMs). In the presence of Cav-1 a “caveolin-induced *L_o_* phase” can be formed, embedded in the first and characterized by d_3_ = 34 and d_4_ = 4.2 Å.

A schematic drawing describing a possible molecular arrangement of WT-DRMs and, in the absence of Cav-1 of KO-DRMs, is reported in Figure 5. We speculate that Cav-1 was inserted vertically in the inner leaflet, as proposed by Liu et al. in a recent molecular dynamics simulation study concerning the interaction of this protein with an asymmetric bilayer mimicking actual lipid-rafts [51]. The insertion of caveolin in the inner leaflet could induce in the “cholesterol-rich *L_o_* phase”, existing in both WT and KO-DRMs (blue and characterized by d_1_ and d_4_), an ordered “caveolin-induced *L_o_* phase” (green and characterized by d_3_ and d_4_). A less ordered *L_d_* phase (red and characterized by d_2_ and d_5_) due to unsaturated lipids is always present. In agreement with biophysical models of lipid-rafts [45,46], cholesterol would be preferentially inserted in the “cholesterol-rich *L_o_* phase”. Thus, in our model even the “caveolin-induced *L_o_* phase” would be rich in cholesterol.

## 5. Conclusions

The results presented in this paper indicate that Cav-1 plays a pivotal role in the constitution of ordered domains in actual lipid bilayers. Both biophysical techniques prompt the hypothesis that occurrence of Cav-1 in the mono- or bilayers induces an ordered molecular organization, namely “caveolin-induced *L_o_* phase”, lacking in the KO-DRMs. Our observations suggest that a self-assembly molecular mechanism could exist, leading to the formation of this phase triggered by the interaction of Cav-1 with the membrane lipid components and cholesterol.

## Supplementary Materials

The following are available online at https://www.mdpi.com/2218-273X/9/7/287/s1, Table S1: Proteome of WT and Cav-1 KO DRMs from mouse lungs; Table S2: Fatty acid composition of DRMs; Figure S1: Cav-1 insertion in protein-free WT DRMs derived liposomes; Figure S2: Characterization of DRMs lipids; Figure S3: X-ray scattering analysis of DRMs from WT and Cav-1 KO mouse adipose tissue.
