# Supplementary material for: Caveolin-1 Endows Order in Cholesterol-Rich Detergent Resistant Membranes
Source: Biomolecules. 2019 Jul 17;9(7):287. doi: 10.3390/biom9070287 (PMC6680987; doi:10.3390/biom9070287)
Supplement: Supplementary file 1 [file biomolecules-09-00287-s001.zip › biomolecules-527669-supplementary/Supplementary_Table S2-Figure S1- S2-S3.pdf]

biomolecules

MDPI

Supplementary Information

Caveolin-1 endows order in cholesterol-rich detergent resistant membranes

Carla Raggi[1†], Marco Diociaiuti[2†], Giulio Caracciolo[3], Federica Fratini[4], Luca Fantozzi[5], Giovanni Piccaro[6], Katia Fecchi[7], Elisabetta Pizzi[4], Giuseppe Marano[7], Fiorella Ciaffoni[4], Elena Bravo[8], Maria L. Fiani[9]* and Massimo Sargiacomo[9]*

[1] National Center for Control and Evaluation of Medicines, Istituto Superiore di Sanità, 00161 Rome, Italy; carla.raggi@iss.it (C.R.)

[2] National Center for Rare Diseases, Istituto Superiore di Sanità, 00161 Rome, Italy; marco.diociaiuti@iss.it (M.D.)

[3] Department of Molecular Medicine, "La Sapienza" University, 00161 Rome, Italy; giulio.caracciolo@uniroma1.it (G.C.)

[4] Scientific Service for Core Facilities, Istituto Superiore di Sanità, 00161 Rome, Italy; federica.fratini@iss.it (F.F.); elisabetta.pizzi@iss.it (E.P.); fiorella.ciaffoni@iss.it (F.C.)

[5] Present address : ARPALAZIO, Via Salaria per L'Aquila 6/8, 02100 Rieti, Italy; luca.fantozzi@arpalazio.gov.it (L.F.)

[6] Notified Body, Istituto Superiore di Sanità, 00161 Rome, Italy; giovanni.piccaro@iss.it (G.P.)

[7] Reference Centre for Gender Medicine, Istituto Superiore di Sanità, 00161 Rome, Italy; katia.fecchi@iss.it (K.F.) ; giuseppe.marano@iss.it (G.M.)

[8] Scientific Service for Research Coordination and Support, Istituto Superiore di Sanità, 00161 Rome, Italy; elena.bravo@iss.it (E.B.)

[9] National Center for Global Health, Istituto Superiore di Sanità, 00161 Rome, Italy ; massimo.sargiacomo@iss.it (M.S.); maria.fiani@iss.it (M.L.F.)

* Authors to whom correspondence should be addressed.

† These authors contributed equally to this work.

	**PC**		**PE**		**SM**	
	**WT**	**KO**	**WT**	**KO**	**WT**	**KO**
**total SFA (%)**	86,88±2,17	85,32±1,34	37,69±3,44	39,1±3,82	74,16±4,44	72,95±5,23
**total MUFA (%)**	7,55±2,40	8,90±2,31	12,47±3,16	14,1±3,90	25,73±4,11	25,60±4,37
**total PUFA (%)**	5,57±3,06	5,78±2,47	49,84±5,96	46,8±6,24	0,11±0,00	1,45±3,55
**total MUFA+PUFA(%)**	13,12±2,03	14,68±1,69	62,31±3,43	60,9±3,83	25,84±4,11	27,05±5,25
**Unsaturation Index (U.I)**	0,32±0,11	0,33±0,07	5,64±1,24	5,1±0,89	0,35±0,08	0,40±0,13
**ω6 (%)**	5,12±2,91	5,52±2,38	20,92±2,28	19,4±3,03	0,00	0,00
**ω3 (%)**	0,39±0,48	0,32±0,29	29,43±4,77	27,3±3,60	0,00	0,00
**ω6/ ω3**	15,29±8,91	22,47±15,86	0,72±0,11	0,7±0,11	-	-
**18:1 (n-9+n-7)**	5,84±1,74	6,6±1,15	-	-	-	-

Table S2. Fatty acid composition of DRMs. Main phospholipid classes (PC, PE) and sphingomyelin (SM) of DRMs from lungs of WT and KO mice were extracted and analyzed by gas chromatography. Percentages of total saturated (SFA), mono (MUFA) and polyunsaturated (PUFA) fatty acids are presented as. mean±s.d. of seven separate experiments (*$p < 0.05$).

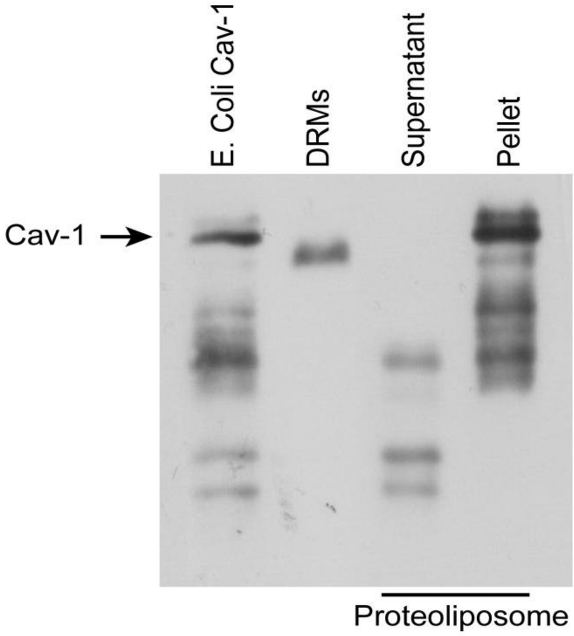

Figure S1. Cav-1 insertion in protein free WT DRMs derived liposomes. Proteoliposomes formation was evaluated for the presence of recombinant His-tagged Cav-1 molecules in the pellet recovered after centrifugation. Western blot analysis for Cav-1 shows from left to right recombinant Cav-1, WT DRM, and Cav-1 containing proteoliposomes. After proteoliposomes centrifugation Cav-1 fragments are differently distributed either in the pellet or in the supernatant according to their affinity for lipids.

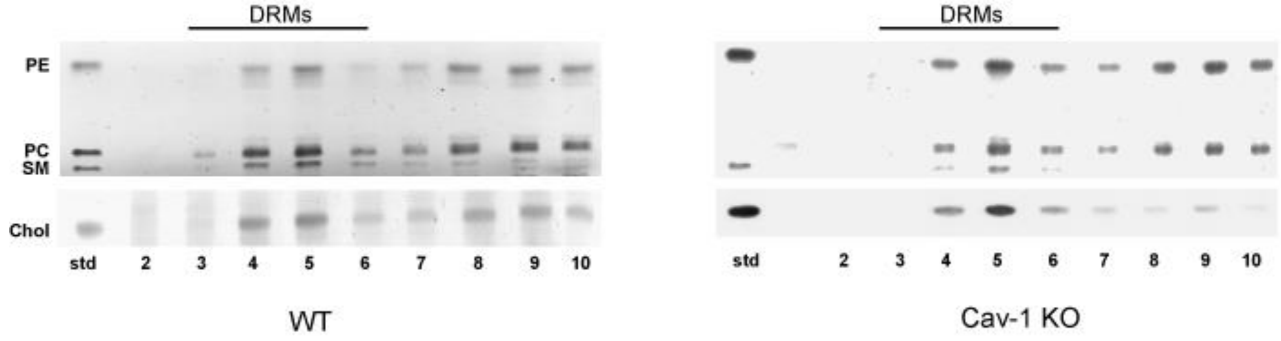

Figure S2. Characterization of DRMs lipids. Post-nuclear homogenates from WT and Cav-1 KO lung tissue were extracted using 1 % TX-100 and fractionated in 5–30 % sucrose gradient. Equal volumes of sucrose gradient fractions were subjected to TLC analysis. Fractions 3-6 corresponding to DRMs showed enrichment of sphingomyelin (SM) and cholesterol (Chol).

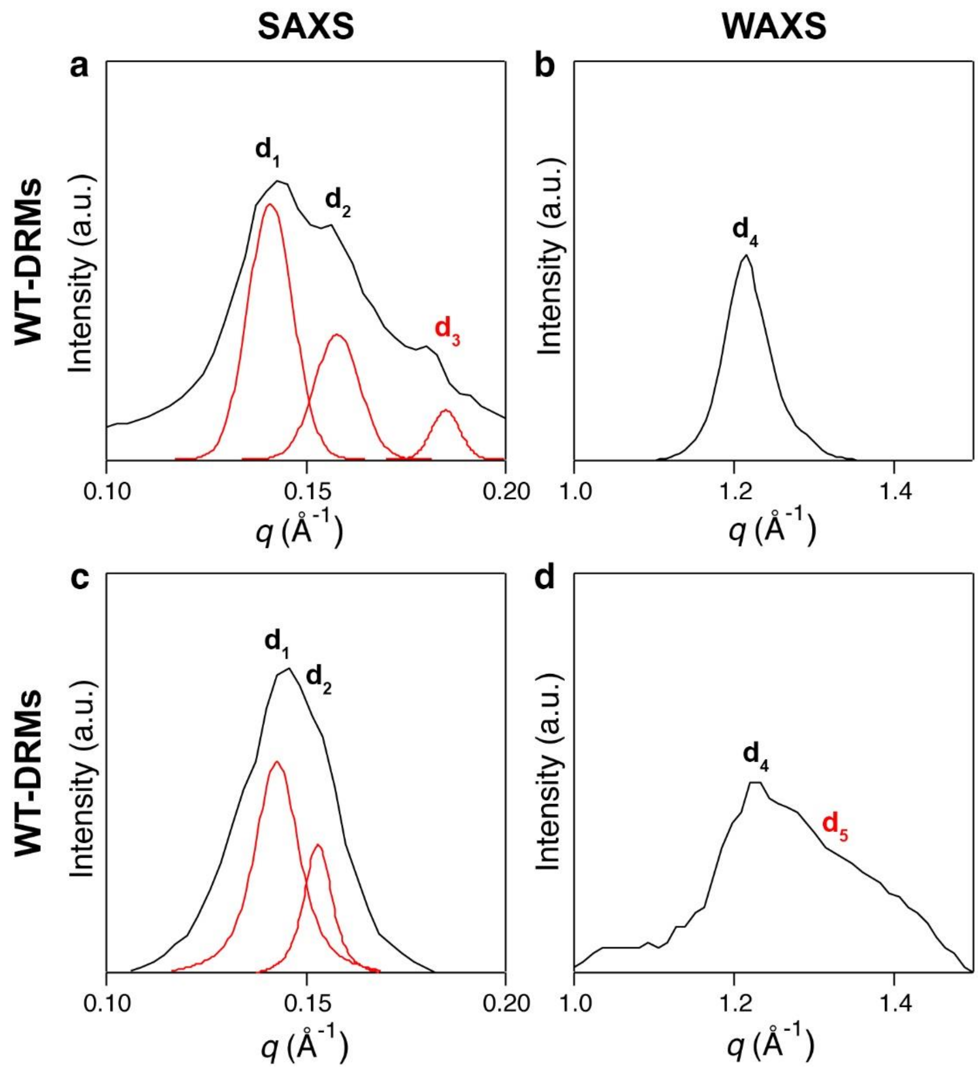

Figure S3. X-ray scattering analysis of DRMs from WT and Cav-1 KO mouse adipose tissue: (a) The SAXS pattern of WT DRMs was dominated by two lamellar phases (d-spacings of d_1 = 43 and d_2 = 39 Å) coexisting with a phase characterized by d_3 = 34.0 Å (symbol in red). (b) The wide-angle X-ray scattering (WAXS) pattern of lung WT-DRMs shows a sharp diffraction peak due to a lipid lattice with repeat spacing of d_4 = 4.2 Å. (c) In the SAXS pattern of lung KO-DRMs, only two diffraction peaks of lamellar phases were observed (d_1 = 43 and d_2 = 39 Å). (d) The WAXS scan of lung KO-DRMs exhibited a double peak (d_4 = 4.2 and d_5 = 3 Å), typical of disordered lipid phases.
